# The Role of IS*CR1*-Borne P_OUT_ Promoters in the Expression of Antibiotic Resistance Genes

**DOI:** 10.3389/fmicb.2018.02579

**Published:** 2018-10-30

**Authors:** Claire Lallement, Cécile Pasternak, Marie-Cécile Ploy, Thomas Jové

**Affiliations:** INSERM, CHU Limoges, RESINFIT, U1092, University of Limoges, Limoges, France

**Keywords:** antibiotic resistance, promoters, IS*CR1*, expression, insertion sequence element

## Abstract

The IS*CR1* (Insertion sequence Common Region) element is the most widespread member of the IS*CR* family, and is frequently present within γ-proteobacteria that occur in clinical settings. IS*CR1* is always associated with the 3′Conserved Segment (3′CS) of class 1 integrons. IS*CR1* contains outward-oriented promoters P_OUT_, that may contribute to the expression of downstream genes. In IS*CR1*, there are two P_OUT_ promoters named P_CR1-1_ and P_CR1-2_. We performed an *in silico* analysis of all publically available IS*CR1* sequences and identified numerous downstream genes that mainly encode antibiotic resistance genes and that are oriented in the same direction as the P_OUT_ promoters. Here, we showed that both P_CR1-1_ and P_CR1-2_ significantly increase the expression of the downstream genes *bla*_CTX-M-9_ and *dfrA19*. Our data highlight the role of IS*CR1* in the expression of antibiotic resistance genes, which may explain why IS*CR1* is so frequent in clinical settings.

## Introduction

**FIGURE 1:**
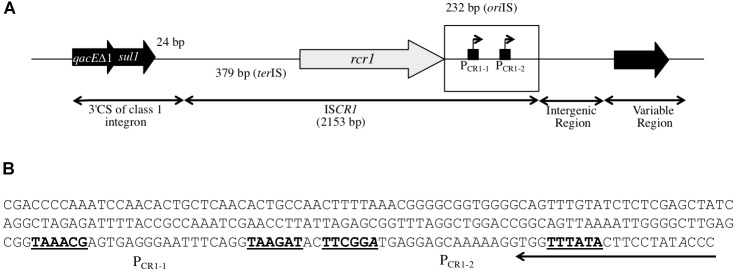
Structure of IS*CR1*. **(A)** Schematic representation of the IS*CR1* element in its genetic context. IS*CR1* is inserted 24 bp downstream of the *sul1* gene (sulfonamide resistance gene) found in the class 1 integrons 3′ Conserved Segment (3′CS). IS*CR1* is flanked by an *ori*IS-(232 bp) and a *ter*IS-containing region (379 bp). It includes a *rcr1* transposase gene, the P*rcr1* promoter for *rcr1* and two putative outward P_CR1-1_ and P_CR1-2_ promoters. A variable region is located downstream IS*CR1*, separated by an intergenic region. The *ori*IS-containing region is boxed. **(B)** Nucleotide sequence of the 232 bp *ori*IS-containing region of IS*CR1*. The *ori*IS sequence that delimitates IS*CR1* is highlighted. The –35 and –10 elements of the putative P_CR1-1_ and P_CR1-2_ promoters are written in bold. Their corresponding transcriptional start sites as previously mapped are indicated in italics.

Antimicrobial resistance is often mediated by the dissemination of antibiotic resistance genes (ARG) that are carried by mobile genetic elements (MGEs) including plasmids, insertion sequences (IS), transposons (Tn) and integron gene cassettes (Partridge, 2011) which are harbored by bacteria across all phyla and environments (Aminov, 2011). In addition, some MGEs may carry promoters that ensure or increase expression of downstream ARG. Several IS including IS*1999*, IS*Ecp1*, IS*Kpn23* (reviewed in Vandecraen et al., 2017) display a complete outwardly oriented functional promoter usually referred as P_OUT_ that enhances expression of downstream ARGs. Other IS like IS*1* or IS*257* only contain the -35 element that generates a hybrid functional promoter when associated with a downstream putative -10 element (Goussard et al., 1991; Simpson et al., 2000). Most often, these IS-borne promoters allow sufficient expression of ARGs to confer the antibiotic resistance phenotype. IS from the IS*CR* family are related to the IS*91* family and display a *rcr* gene encoding a putative RCR transposase belonging to the ubiquitous HUH endonuclease superfamily (Chandler et al., 2013). HUH transposases of the IS*91* family catalyze the transposition of their cognate IS by the rolling-circle replication of the element from one boundary, named *ori*IS, to the other referred to as the *ter*IS (Tavakoli et al., 2000; del Pilar Garcillán-Barcia et al., 2001; Yassine et al., 2015). However, so far, there is no experimental evidence for transposition of any of the IS*CR* elements. Four out of the fifteen members of the IS*CR* family are commonly found in γ-proteobacteria, namely IS*CR1*, IS*CR2*, IS*CR3* and IS*CR5*, and IS*CR1* predominates in strains isolated in clinical settings (Toleman et al., 2006). IS*CR1* was first identified as a conserved region disrupting the 3′ conserved segment (3′CS) of class 1 integrons (Figure 1A; Stokes et al., 1993). The region downstream of IS*CR1* (*ori*IS side) is variable and often associated with ARG (Arduino et al., 2002; Toleman et al., 2006; Rodríguez-Martínez et al., 2007; Wachino et al., 2011). Previous studies identified the presence of two putative promoters located on the *ori*IS side of the IS*CR1*, namely P_CR1-1_ and P_CR1-2_, suggesting that IS*CR1* could impact the expression of downstream genes (Figure 1B; Mammeri et al., 2005; Rodriguez-Martinez et al., 2006). To assess the potential function of these promoters in the expression of downstream genes, we first performed an extensive *in silico* analysis of all IS*CR1* sequences publically available (GenBank^®^) to determine the diversity of putative downstream ARGs. Here, we show experimentally by means of a reporter gene assay that IS*CR1* directly contributes to the expression of different ARGs via these two P_OUT_ promoters.

## Materials and Methods

### Bacterial Strains and Plasmids

Bacterial strains and plasmids are listed in (Supplementary Table S1). Bacterial strains were grown in Lysogeny Broth (LB) broth at 37°C. Media were supplemented with kanamycin (25 μg/mL) when required.

### Plasmid Constructions

We used the reporter plasmid pSU38Δtot*lacZ* (Jové et al., 2010) and three derived plasmids in which IS*CR1* and/or regions adjacent to IS*CR1* were inserted in transcriptional fusion with the reporter gene *lacZ*. Fragments of IS*CR1* and/or regions adjacent to IS*CR1* were amplified from two *Salmonella enterica* subsp. *enterica* strains carrying IS*CR1* followed by either *bla*_CTX-M-9_, or *dfrA19* genes (Espéli et al., 2001) as they belong to the most prevalent antibiotic resistance gene families found in the variable region downstream IS*CR1*. Primers (Sigma-Aldrich^®^) used for cloning are listed in Supplementary Table S2. For each construction, amplifications were performed using the Phusion^®^ Polymerase (Thermo Fisher Scientific). PCR products were loaded and visualized by means of a 0.8% agarose gel, extracted and purified with the Wizard^®^ SV Gel and PCR Clean-Up System (Promega, Madison, WI, United States). PCR products were cloned into the EcoRI and BamHI unique restriction sites of pSU38Δtot*lacZ*. Transformants were selected on LB medium supplemented with kanamycin. Recombinant plasmids were verified by PCR with primers targeting the insert and by sequencing.

### β-Galactosidase Assays

β-galactosidase assays were performed in the *E. coli* MG1656 strain (Supplementary Table S1; Espéli et al., 2001) as previously described (Miller, 1993) for nine independent assays for each construct.

### Minimum Inhibitory Concentration (MIC) Determination

Minimum inhibitory concentrations were performed by the microdilution method in Mueller-Hinton broth in three independent experiments as recommended by the French Antibiogram committee guidelines^1^.

### Statistical Analysis

Statistical analyses were performed using the Mann-Whitney test with two paired groups.

### GenBank^®^ IS*CR1* Element Sequence Analysis

The amino acid sequence of the RCR1 transposase encoded by IS*CR1* (accession number CAJ84008) was blasted with BLASTp (NCBI). The matching sequences were filtered to retain RCR1 peptide sequences with an amino acid identity level (equal or) higher than 98%. Corresponding nucleotide sequences in which the *ori*IS region was partial or truncated were discarded. The remaining nucleotide sequences were sorted out in 93 groups according to the nature of the first gene adjacent to IS*CR1*: non-annotated nucleotide sequences with identified open reading frame (ORF) longer than 150 bp were included into the analysis. To define a novel gene group, we used a cut-off of 95% amino acid identity of the encoded protein, except for antimicrobial resistance genes (ARGs) for which a single amino-acid variation was used as threshold for inclusion into a group. Finally, one nucleotide sequence representing each gene group was submitted to blastN to identify previously non-annotated nucleotide sequences (only 100% identical sequences were kept). This data extraction was performed on 2017-01-19.

### Quantification of *bla*_CTX-M-9_ and *dfrA19* Transcripts

Total RNA was extracted with the NucleoSpin^®^ RNA Extraction Kit (Macherey-Nagel Inc.). Contaminating DNA was removed from RNA samples by using the Turbo DNA-free Kit (Ambion). cDNAs were synthesized from 1 μg of DNase-treated total RNA by using PrimeScript^TM^ RT Reagent kit (TaKaRa Clontech). cDNA was quantified by PerfeCTa^®^ SYBR^®^ Green FastMix^®^ Kit (Quanta BioSciences^TM^) with adequate oligonucleotides (Supplementary Table S2). Three independent experiments were performed, each in triplicate. Relative expressions of the *bla*_CTX-M-9_ (Primers 16 and 17) and *dfrA19* (Primers 18 and 19) genes were estimated by normalizing transcript copy number to those of the housekeeping gene *rpoB* (Primer 20 and 21). The impact of IS*CR1 ori*IS has been calculated as ratio between the relative expression of each gene in presence and in absence of the IS*CR1 ori*IS.

## Results and Discussion

### Diversity of the IS*CR1* Downstream Genes

In this study, 1127 distinct sequences containing the IS*CR1* element extracted from GenBank^®^ were analyzed *in silico.* The majority of these sequences was recovered from γ-proteobacteria (99.9%) while others were found to be present in uncultured bacteria (*n* = 4) (Supplementary Table S3). All IS*CR1* sequences in this study were associated with the 3′ CS region of class 1 integrons (left-hand side, Figure 1). In contrast, the downstream region of IS*CR1* was identified to be very variable (right-hand side, Figure 1). Interestingly, a large percentage of the analyzed IS*CR1* elements (*n* = 946, 84%) carried an adjacent gene oriented in the same direction as the *rcr1* encoding transposase gene (top strand). This suggests that these genes might be expressed from the IS*CR1* P_OUT_ promoters (Figure 1). The functions of these top strand genes adjacent to IS*CR1* fell into three categories (Supplementary Table S4). The most represented genes (*n* = 429) encoded truncated insertion sequence transposases, most often IS*Ec28* (*n* = 418), more rarely IS*Ec29*, IS*Aba125* or IS*Ecp1* (Supplementary Table S4). The second most important group (*n* = 379) was identified as known or putative ARGs encoding resistance to five families of antibiotics: trimethoprim (*n* = 125), β-lactams (including extended-spectrum β-lactamase, ESBL, genes) (*n* = 121), quinolones (*n* = 113), chloramphenicol (*n* = 12), and aminoglycosides (*n* = 8). For each antibiotic family, different genes or alleles were identified, that may lead to different resistance phenotypes (Supplementary Table S4). The last group (*n* = 138) includes genes involved in other cellular process or genes of unknown function (Supplementary Table S4).

### IS*CR1* Contributes to the Downstream Expression of ARGs via Its P_OUT_ Promoters

**FIGURE 2:**
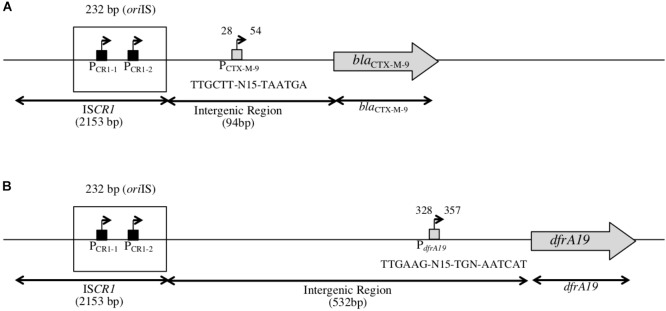
Structure of the genetic context of *bla*_CTX-M-9_ and *dfrA19* genes downstream of IS*CR1*. **(A)** Schematic representation of the genetic context of *bla*_CTX-M-9_ as found in our *in silico* analysis, located 94 bp away from IS*CR1* (accession number: AF174129). P_CTX-M-9_ indicates a putative promoter for *bla*_CTX-M-9_ located in the intergenic region, 28 bp away from IS*CR1*. **(B)** Schematic representation of the genetic context of *dfrA19* as found in our *in silico* analysis, located 532 bp away from IS*CR1* (accession number: AM234698). *dfrA19* indicates a putative promoter for *dfrA19* located in the intergenic region, 328 bp away from IS*CR1*.

**FIGURE 3:**
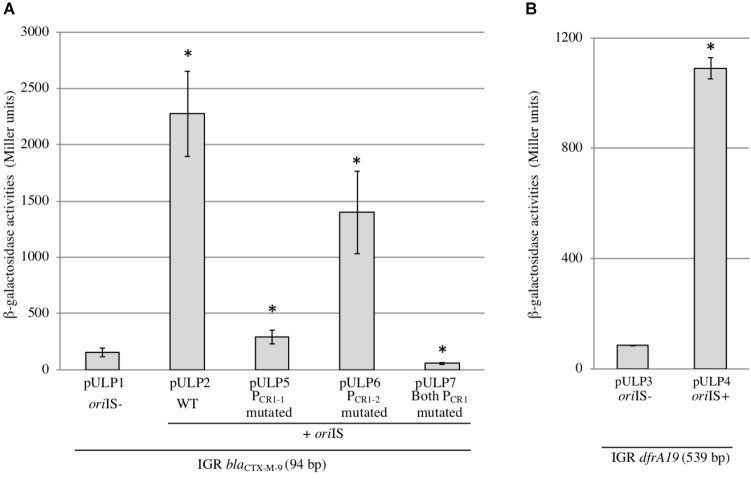
Activities and characterization of the IS*CR1* P_OUT_ promoters. β-galactosidase activities were measured from *lacZ*-transcriptional fusions with the intergenic region (IGR) cloned either in absence (*ori*IS–) or in presence (*ori*IS+) of the 232 bp long IS*CR1 ori*IS region. The genes tested were *bla*_CTX-M-9_ **(A)** and *dfrA19* **(B)**. Derivatives in which one or both P_CR1_ promoters were mutated were also tested for *bla*_CTX-M-9_ **(A)**. Constructions are described in (Supplementary Table S1). The results are the average of at least three independent experiments. ^∗^*p* < 0.001.

To investigate the impact of P_OUT_ promoters on the expression of downstream genes, we focused on the two following ARGs: *bla*_CTX-M-9_ (conferring an Extended-Spectrum Beta-Lactamase resistance phenotype) (accession number: AM234698) and *dfrA19* (resistance to trimethoprim) (accession number: AF174129), which were the ARG sequences most frequently found in our *in silico* analysis. Their coding sequences are located 94 and 532 bp away from the *ori*IS, respectively (Figure 2); this distance is thereafter referred as to the intergenic region or IGR. We cloned each IGR in front of the promoter-less *lacZ* gene in absence or in presence of the IS*CR1 ori*IS region (that contains the P_OUT_ promoters) and compared the resulting β-galactosidase activities. When the *lacZ* coding sequence was preceded by each IGR alone, the level of β-galactosidase activity ranged from 84 MU (Miller Units) to 155 MU for *dfrA19*, and *bla*_CTX-M-9,_ respectively (Figure 3A, pULP1 and Figure 3B, pULP3). These results indicate the presence of a functional promoter in each IGR. Accordingly, a conserved σ^70^ promoter sequence was identified in the IGR of *bla*_CTX-M-9_ (TTGCTT-N_15_-TAATGA) and *dfrA19* (TTGAAG-N_15_-TGN-AATCAT), which could account for the observed β-galactosidase expression (Figure 2). However, when both the *ori*IS and the IGR were present, the β-galactosidase activity was enhanced by 13- and 15-fold for *dfrA19* and *bla*_CTX-M-9_, respectively (Figure 3A, pULP2 and Figure 3B, pULP4). These results indicate that the IS*CR1 ori*IS region significantly increases the expression level of downstream genes and confirm that IS*CR1* harbors a functional P_OUT_ promoter. We thus showed that both IS*CR1 ori*IS and IGR are involved in gene expression of *dfrA19* and *bla*_CTX-M-9_. However, as we observed in our *in silico* analysis (Supplementary Table S4), the sequence and length of the IGR vary from 0 to 1211 bp. Further analysis with other IS*CR1* elements with different downstream genes are needed to elucidate to which extent the sequence of the IGR contribute to downstream gene expression.

To determine whether the positive effect of the IS*CR1 ori*IS on the expression of the downstream gene relies on the two P_OUT_ promoters, P_CR1-1_ (TAAACG-N_17_-TAAGAT) and P_CR1-2_ (TTCGGA-N_18_-TTTATA), we constructed derivatives of pULP2 (*ori*IS-IGR_CTX-M-9_-*lacZ*) in which the putative -10 element, of either P_CR1-1_ or P_CR1-2_ or both promoters was mutated. Mutation of P_CR1-1_ (pULP5) or P_CR1-2_ (pULP6) reduced by 85 and 40%, respectively, the overall β-galactosidase activity compared to the pULP2 wild-type construction (Figure 2A). Concomitant mutations of both P_CR1-1_ and P_CR1-2_ dropped the expression to the basal level detected in absence of *ori*IS (Figure 3A, pULP7 versus pULP1). These results indicated that both P_CR1-1_ and P_CR1-2_ are functional and, together, are responsible for the contribution of IS*CR1* to the expression of downstream top strand genes. Consistently, several earlier reports mapped transcriptional START sites in the *ori*IS that are compatible with the P_CR1-1_ and P_CR1-2_ (Mammeri et al., 2005; Rodriguez-Martinez et al., 2006). P_CR1-1_ also appears to be stronger than P_CR1-2_ in agreement with its higher conservation degree of its -10 hexamer, with respect to the σ70 consensus sequence (four bases out of six versus three for P_CR1-2_). These tandem promoters display a synergistic effect but the exact underlying mechanisms remain to be elucidated.

### The IS*CR1 ori*IS Is Required to Confer the *bla*_CTX-M-9_-Mediated Resistance

To assess whether the increased level of gene expression due to the IS*CR1 ori*IS region has a phenotypic impact, we measured the level of resistance conferred by *dfrA19* and *bla*_CTX-M-9_ genes in absence or in presence of the *ori*IS region in *Escherichia coli*. For this purpose, each gene was cloned in pSU38Δtot*lacZ* (Supplementary Table S1) with its own IGR preceded or not by the *ori*IS region. Subsequently, we determined the MIC of the respective clones in presence of the corresponding antibiotic (cefotaxime or trimethoprim) (see below). The *Escherichia coli* MG1655*lac-*strain harboring the empty plasmid (pSU38Δtot*lacZ*) was susceptible to both cefotaxime (MIC < 0.5 μg/mL) and trimethoprim (MIC < 4 μg/mL). When MG1655*lac-* is transformed with a pSU38Δtot*lacZ* derivative that harbors the *bla*_CTX-M-9_ coding sequence alone (pULP13) or preceded by its own IGR (pULP12), the MIC for cefotaxime was also <0.5 μg/mL. The MIC for cefotaxime significantly increased in pULP11 which contains both IGR and *ori*IS region (MIC > 512 μg/mL). These findings demonstrated that the IS*CR1 ori*IS region is required for *bla*_CTX-M-9_ to confer a cefotaxime resistant phenotype, most likely mediated by the activity of P_CR1-1_ and P_CR1-2_. We performed quantification of transcripts and showed that the *bla*_CTX-M-9_ transcript number increased by 99-fold (98.82 ± 8.29) in presence of IGR. These results correlate with MIC findings.

In contrast, when preceded by its IGR, the level of trimethoprim resistance conferred by *dfrA19* was similar in absence or in presence of the *ori*IS region (pULP08 MIC = > 2048 μg/mL, pULP09 MIC = > 2048 μg/mL), while the *dfrA19* coding sequence alone did not confer any resistance (pULP10, MIC < 4 μg/mL). Susceptibility to higher concentrations of trimethoprim could not be determined since they excess its solubility in DMSO. The *dfrA19* resistance gene confers a higher level of resistance compared to other *dfrA* alleles, such as *dfrA10* for example (MIC: 500 μg/mL) (Parsons et al., 1991). We observed a similar resistance phenotype (trimethoprim MIC = > 2048 μg/mL) in absence or in presence of the *ori*IS region. These results were surprising according to β-galactosidase results obtained with or without the IGR sequence (Figure 3). Quantification of transcripts of *dfrA19* confirmed that the IGR plays a role in the expression of the gene. Indeed, we obtained a 20-fold change of transcript number (20.03 ± 4.1, *dfrA19*) in presence of IGR. Such dissociation between the resistance phenotype and the level of gene expression has been previously described (Barraud and Ploy, 2015). Furthermore, this might also be explained by the nature of the enzyme encoded by *dfr* genes. Indeed, DFR enzymes are insensitive toward trimethoprim, so neither a high concentration of antibiotic nor the quantity of DFR enzymes will affect the level of resistance. Little is known about the *dfrA19* gene and noticeably, it seems to only occur associated with IS*CR1* element.

## Conclusion

Our data highlight the functionality of the two P_OUT_ promoters carried by the IS*CR1* element. The fact that those two functional P_OUT_ promoters contribute to the expression of various downstream genes, including ARG may explain why IS*CR1* is so frequent in clinical settings. Indeed, IS*CR1* gives an advantage to the bacteria for antimicrobial resistance expression and one can hypothesize that antibiotic selective pressure has promoted the selection of IS*CR1*-carrying bacteria.

## Author Contributions

M-CP and TJ conceived the study. TJ coordinated the study. CL, TJ, and CP performed the experiments. All authors analyzed the data and wrote the manuscript.

## Conflict of Interest Statement

The authors declare that the research was conducted in the absence of any commercial or financial relationships that could be construed as a potential conflict of interest.

## Footnotes

1 http://www.sfm-microbiologie.org/UserFiles/files/casfm/CASFMV1_0_MARS_2017.pdf
