# Supplementary material for: The Role of ISCR1-Borne POUT Promoters in the Expression of Antibiotic Resistance Genes
Source: Front Microbiol. 2018 Oct 30;9:2579. doi: 10.3389/fmicb.2018.02579 (PMC6218425; doi:10.3389/fmicb.2018.02579)
Supplement: Supplementary file 1 [file Table_1.DOCX]

**Table S1:** Strains and plasmids used in this study

| **Strains/plasmids** | **Genotype or description** | **Source or reference** |
| --- | --- | --- |
| Bacterial Strains |  |  |
| *Salmonella enterica* subsp. *enterica* serovar Montevideo C1 | Clinical isolate carrying  IS*CR1*-*bla*_CTX-M-9_ | Laboratory collection |
| *Salmonella enterica* subsp. *enterica* serovar Keurmassar | Clinical isolate carrying  IS*CR1*-*dfrA19* | (Garnier et al., 2006) |
| *Escherichia coli* K-12 MG1655*lac-* |  | (Espéli et al., 2001) |
| **Plasmids** |  |  |
| pSU38Δtot*lacZ* | Vector carrying *lacZ* coding sequence with no promoter. | (Jové et al., 2010) |
| pULP1 | 94bp IGR*bla*_CTX-M-9_ cloned into pSU38Δtot*lacZ* (primers 2 and 3 from isolate C1). | This study |
| pULP2 | *ori*IS and 94bp IGR*bla*_CTX-M-9_ cloned into pSU38Δtot*lacZ* (primers 1 and 3 from isolate C1). | This study |
| pULP3 | 532bp IGR*dfrA19* cloned into pSU38Δtot*lacZ* (primers 4 and 5 from *S.* Keurmassar). | This study |
| pULP4 | *ori*IS 532bp IGR*dfrA19* cloned into pSU38Δtot*lacZ* (primers 1 and 5 from isolate Keurmassar). | This study |
| pULP5 | pULP2 mutated with primers 6 and 7 | This study |
| pULP6 | pULP2 mutated with primers 8 and 9 | This study |
| pULP7 | pULP6 mutated with primers 8 and 9 | This study |
| pULP8 | *ori*IS, 532bp IGR*dfrA19* and *dfrA19* cloned into pSU38Δtot*lacZ* (primers 15 and 13 from isolate Keurmassar). | This study |
| pULP9 | 532bp IGR*dfrA19* and *dfrA19* cloned into pSU38Δtot*lacZ* (primers 14 and 13 from isolate Keurmassar). | This study |
| pULP10 | *dfrA19* cloned into pSU38Δtot*lacZ* (primers 12 and 13 from isolate Keurmassar) | This study |
| pULP11 | *ori*IS, 94bp IGR*bla*_CTX-M-9_ and *bla*_CTX-M-9_  cloned into pSU38Δtot*lacZ* (primers 1 and 10 from isolate C1). | This study |
| pULP12 | IGR*bla*_CTX-M-9_ and *bla*_CTX-M-9_ cloned into pSU38Δtot*lacZ* (primers 5 and 10 from isolate C1). | This study |
| pULP13 | *bla*_CTX-M-9_  cloned into pSU38Δtot*lacZ* (primers 11 and 10 from isolate C1) | This study |

|  |  |  |
| --- | --- | --- |
