# Supplementary material for: The Role of ISCR1-Borne POUT Promoters in the Expression of Antibiotic Resistance Genes
Source: Front Microbiol. 2018 Oct 30;9:2579. doi: 10.3389/fmicb.2018.02579 (PMC6218425; doi:10.3389/fmicb.2018.02579)
Supplement: Supplementary file 2 [file Table_2.DOCX]

**Table S2: Primers used in this study**

| **Primers** | **Sequence (5’-3’)** | | | |
| --- | --- | --- | --- | --- |
| 1 | CCGGAATTCCGACCCCAAATCCAACACTG |  |  |  |
| 2 | CCGGAATTCGAGGCGCGACAGAAAAATCG |  |  |  |
| 3 | CGCGGATCCATCATCCGTTGCACTCTCT |  |  |  |
| 4 | CCGGAATTCAGTAGTGTCCCCTGTCGGTTTG |  |  |  |
| 5 | CGCGGATCCTCAAGTTGTGGGTGACTCATTAAAACTCCAAACCAGTCG |  |  |  |
| 6 | GTGAGGGAATTTCAGGCGAGGCACTTCGGATGAGGAGCAAAAAGG |  |  |  |
| 7 | GCTCCTCATCCGAAGTGCCTCGCCTGAAATTCCCTCACTCGTTTAC |  |  |  |
| 8 | TGAGGAGCAAAAAGGTCGCTGCTACTTCCTATACCCGAGGCGCGAC |  |  |  |
| 9 | GCGCCTCGGGTATAGGAAGTAGCAGCGACCTTTTTGCTCCTCATCC |  |  |  |
| 10 | GCGGGATCCTTACAGCCCTTCGGCATGATT |  |  |  |
| 11 | CGCGAATTCATGGTGACAAAGAGAGTGCA |  |  |  |
| 12 | GCTAGGATCCATGAGTCACCCACAACTTG |  |  |  |
| 13 | TGCACTGCAGTTCTCGAGCCAATAATG |  |  |  |
| 14 | GATAGGATCCAGTAGTGTCCCCTGTCG |  |  |  |
| 15 | TTCAGGATCCGACCCCAAATCCAACACTG |  |  |  |
