# Supplementary material for: The Role of ISCR1-Borne POUT Promoters in the Expression of Antibiotic Resistance Genes
Source: Front Microbiol. 2018 Oct 30;9:2579. doi: 10.3389/fmicb.2018.02579 (PMC6218425; doi:10.3389/fmicb.2018.02579)
Supplement: Supplementary file 3 [file Table_3.DOCX]

**Table S3: Bacterial genera in which complete IS*CR1* along with its adjacent genes were recovered (n=1127, 19/01/2017).**

| **Bacterial genus** | **Number of hits** | | | |
| --- | --- | --- | --- | --- |
| *Acinetobacter* | 346 |  |  |  |
| *Klebsiella* | 235 |  |  |  |
| *Enterobacter* | 212 |  |  |  |
| *Escherichia* | 150 |  |  |  |
| *Salmonella* | 36 |  |  |  |
| *Vibrio* | 26 |  |  |  |
| *Pseudomonas* | 20 |  |  |  |
| *Citrobacter* | 17 |  |  |  |
| Autres | 17 |  |  |  |
| *Proteus* | 16 |  |  |  |
| *Serratia* | 13 |  |  |  |
| *Providencia* | 11 |  |  |  |
| *Aeromonas* | 10 |  |  |  |
| *Shigella* | 6 |  |  |  |
| *Shewanella* | 6 |  |  |  |
| *Morganella* | 6 |  |  |  |
