# Supplementary material for: The Role of ISCR1-Borne POUT Promoters in the Expression of Antibiotic Resistance Genes
Source: Front Microbiol. 2018 Oct 30;9:2579. doi: 10.3389/fmicb.2018.02579 (PMC6218425; doi:10.3389/fmicb.2018.02579)
Supplement: Supplementary file 4 [file Table_4.DOC]

**Table S4**: Diversity of genes located of the top strand downstream IS*CR1* (n=946). Genes are classified according to the function of the encoded protein. Results are detailed for antibiotic resistance genes (n= 379).

| **Function (strand)** | **Gene** | **Distance to IS*CR1* (bp)** | **Number (%) of sequences** |
| --- | --- | --- | --- |
| Insertion sequences |  | 0-229 | 429 (45.3%) |
| Other or unknown functions |  | 7-1211 | 138 (14.6%) |
| Resistance to antibiotics |  | 24- 983 | 379 (40.1%) |
| ***Resistance to trimethoprim*** |  |  | **125 (13.2%)** |
|  | *dfrA3b* | 123 | 9 |
|  | *dfrA10* | 149 | 14 |
|  | *dfrA19* | 494-534 | 101 |
|  | *dfrA24* | 261 | 1 |
| ***Resistance to β-lactams*** |  |  | **121 (12.8%)** |
|  | *bla*_CMY-9_ | 114 | 5 |
|  | *bla*_CMY-11_ | 182 | 1 |
|  | *bla*_CTX-M-1_ | 94 | 1 |
|  | *bla*_CTX-M-2_ | 266 | 47 |
|  | *bla*_CTX-M-9_ | 94 | 25 |
|  | *bla*_CTX-M-14_ | 94-377 | 2 |
|  | *bla*_CTX-M-131_ | 266 | 1 |
|  | *bla*_CTX-M-165_ | 266 | 2 |
|  | *bla*_PER-1_ | 80-126 | 26 |
|  | *bla*_PER-4_ | 90-123 | 3 |
|  | *bla*_PER-7_ | 92-93 | 8 |
| ***Resistance to quinolones*** |  |  | **113 (11.9%)** |
|  | *qnrA1* | 24-166 | 104 |
|  | *qnrA3* | 24-77 | 5 |
|  | *qnrVC6* | 30 | 3 |
| ***Resistance to chloramphenicol*** |  |  | **12 (1.3%)** |
|  | *catA2* | 500-983 | 8 |
|  | *putative catA* | 528 | 3 |
|  | *floR* | 403 | 1 |
| ***Resistance to aminoglycosides*** |  |  | **8 (0.8%)** |
|  | *aphA6* | 140-258 | 7 |
|  | *rmtB* | 120 | 1 |
